# DNA methylation landscape of triple-negative ductal carcinoma *in situ* (DCIS) progressing to the invasive stage in canine breast cancer

**DOI:** 10.1038/s41598-020-59260-4

**Published:** 2020-02-12

**Authors:** Megan Beetch, Sadaf Harandi-Zadeh, Tony Yang, Cayla Boycott, Yihang Chen, Barbara Stefanska, Sulma Mohammed

**Affiliations:** 1 Food, Nutrition & Health Program, Faculty of Land and Food Systems, University of British Columbia, Vancouver, Canada; 2 Department of Comparative Pathobiology, Purdue University, West Lafayette, IN USA; 3 Purdue University Center for Cancer Research, Purdue University, West Lafayette, IN USA

**Keywords:** DNA methylation, Breast cancer, Breast cancer

## Abstract

Triple-negative breast cancer (TNBC) is a subtype of breast cancer unresponsive to traditional receptor-targeted treatments, leading to a disproportionate number of deaths. Invasive breast cancer is believed to evolve from non-invasive ductal carcinoma *in situ* (DCIS). Detection of triple-negative DCIS (TN-DCIS) is challenging, therefore strategies to study molecular events governing progression of pre-invasive TN-DCIS to invasive TNBC are needed. Here, we study a canine TN-DCIS progression and investigate the DNA methylation landscape of normal breast tissue, atypical ductal hyperplasia (ADH), DCIS and invasive breast cancer. We report hypo- and hypermethylation of genes within functional categories related to cancer such as transcriptional regulation, apoptosis, signal transduction, and cell migration. DNA methylation changes associated with cancer-related genes become more pronounced at invasive breast cancer stage. Importantly, we identify invasive-only and DCIS-specific DNA methylation alterations that could potentially determine which lesions progress to invasive cancer and which could remain as pre-invasive DCIS. Changes in DNA methylation during TN-DCIS progression in this canine model correspond with gene expression patterns in human breast tissues. This study provides evidence for utilizing methylation status of gene candidates to define late-stage (DCIS and invasive), invasive stage only or DCIS stage only of TN-DCIS progression.

## Introduction

Breast cancer is classified into subtypes based on the expression of growth factor receptors including the estrogen receptor (*ER*), the progesterone receptor (*PR*), and the receptor for human epidermal growth factor (*HER-2*)^1^. Growth of breast tumors expressing any of these receptors may be controlled effectively by treatment in the adjuvant setting with receptor-targeted drugs^2^. However, breast tumors that do not express any of these receptors have no known effective adjuvant treatment capable of controlling tumor growth. Such tumors are referred to as triple-negative breast cancers (TNBC) and are the most aggressive and lethal of all breast malignancies^2^. TNBC accounts for 15% of breast cancer cases and a disproportionate percentage of breast cancer deaths among women^3^. It has been shown that patients with TNBC have poor prognosis and shorter median time to relapse compared to patients with other subtypes of breast cancer^4^.

Ductal carcinoma *in situ* (DCIS) is defined as a non-invasive overgrowth of cells characterized by high proliferation within the breast ductal system. Studies suggest that triple-negative DCIS (TN-DCIS), a rare type of DCIS, is a precursor stage of invasive breast cancer^5,6^. Therefore, early detection of TN-DCIS is important in preventing breast cancer cases that may progress to triple negative invasive carcinoma. However, TN-DCIS is challenging to detect at early stage in humans^7^. Despite efforts to use immunohistochemistry to measure receptor expression in scientific studies of human DCIS tissues, detection of receptor status, including *ER*, is not routinely implemented in molecular testing of DCIS in clinic settings^8^. Several studies have established the importance of early detection of breast cancer calcification via mammography as part of breast cancer diagnoses^9,10^. However, Kojima and collegues reported that abnormal breast calcifications were only detected in 22% of TN-DCIS compared to 59–72% of other types of DCIS cases, emphazising the challenge associated with detecting this specific breast cancer subtype during its non-invasive term^7^. Furthermore, the high proliferation rate of TN-DCIS accounts for another challenge in detecting TN-DCIS at early stages^7^. It has been suggested that TN-DCIS-derived tumors proliferate twice as fast as luminal A and three times faster than HER2-positive tumors, increasing its metastatic potential to other tissues^11^. Indeed, these unique molecular events have hindered early detection of TNBC and have also limited the prospect of preventing this lethal cancer.

There are many shared features of human and canine breast cancer. As in women and unlike rodent models, the mammary glands are the most common site of cancer in unspayed female dogs^12,13^. In addition, canine models develop invasive mammary tumors faster and have shorter survival times compared to humans, making them an excellent model to study human breast cancer^12,14^. Pre-invasive lesions, such as atypical ductal hyperplasia (ADH) and DCIS, develop spontaneously and naturally before invasive cancer in canine mammary tissue as well^15^. Dogs and humans share many of the same breast cancer risk factors including aging, progesterone exposure, obesity in early life, poor diet, and mutations in *BRCA* genes^12,16,17^. They also undergo similar treatments against breast cancer. Therefore, we have utilized companion dogs to track and molecularly characterize canine TN-DCIS-derived invasive breast cancer.

We have previously shown that canine DCIS and invasive cancer resemble human DCIS and its invasive stage with respect to histopathology, expression of many tumor markers including *ER*, *PR*, *HER2*, and *Ki-67*, and their association with clinical outcomes and imaging characteristics^18–20^. In addition, strong similarities exist between humans and dogs regarding tumor-infiltrating lymphocytes (TIL), such as the relationships between TIL numbers and mammary tumor aggressiveness, between the CD4^+^/CD8^+^ T cell ratio and survival rate, and between Treg cell numbers and poor prognostic factors^21^. Given the many shared features of canine and human breast cancer and the high homology between the canine and human genome, studying companion dogs offers an outstanding opportunity to examine TNBC biology.

Epigenetic alterations within all three components of the epigenome such as DNA methylation, histone covalent modifications, and noncoding RNA mechanisms (including microRNAs) have been reported in canine cancers^22–24^. Promoter hypermethylation can result in gene silencing, and it is an early event in neoplastic progression through transcriptional silencing of tumor suppressor genes^25^. A study that was designed to evaluate changes in promoter CpG island (CGI) methylation status during breast cancer progression from pre-invasive lesions, ADH and DCIS, to invasive ductal carcinoma (IDC) showed that promoter CGI methylation changed significantly in pre-invasive lesions, and was similar in invasive breast cancer and DCIS, suggesting that CGI methylation of tumor suppressor genes is an early event in breast cancer progression^26^. Global hypomethylation also contributes to multi-step carcinogenesis by activating transcription of repetitive sequences and transposable elements, which consequently contributes to genome rearrangements and chromosomal instability in cancer, including breast cancer^27^. Additionally, loci-specific hypomethylation can lead to the activation of oncogenes and pro-metastatic genes^28–31^.

Limited studies have investigated gene-specific changes in DNA methylation in canine breast cancer. DNA methylation patterns in CGIs of *ESR1*, encoding for ERα, and *BRCA1*, an important tumor suppressor gene, have recently been examined^32,33^, reporting changes in DNA methylation of these genes in malignant canine tumors. Moreover, *LINE-1* methylation in cell free DNA (cfDNA) from liquid biopsies was used in a comparative approach of canine and human breast tumors^34^. LINE-1 is a transposable element whose methylation has classically been used as in indicator of changing DNA methylation patterns in human cancer models. Lee and collegues determined methylation levels at *LINE-1* in cfDNA in dogs with benign and malignant breast tumors. Hypomethylation of these elements robustly differentiated canine breast tumors from normal breast tissue. As such, the cut-off level of *LINE-1* methylation based on canine data for distinguishing normal breast tissue from breast tumors was implemented in human cfDNA^34^. This approach successfully predicted the presence of human breast tumors. Studies assessing loci-specific DNA methylation alterations in other cancer types have also been informative. Canine *DLC1* is a critical tumor suppressor gene in many types of cancer. As in human non-Hodgkin’s lymphoma (NHL), the promoter CpG island of *DLC1* in canine NHL is abnormally hypermethylated, relative to healthy lymphoid tissue^35^. Furthermore, as in human, global hypomethylation as determined using restriction patterns of MspI and HpaII enzymes, was found to be a feature of neoplastic cells in the majority of both canine and human lymphoma cases. This confirms that dysregulation of the DNA methylating machinery plays a role in malignant transformation of lymphoid cells in humans and dogs as well^36^. Thus, these studies further support the use of companion dogs as comparative models of human cancer.

In the present study, reduced representation bisulfite sequencing (RRBS) of micro-dissected cells from canine normal breast, ADH, TN-DCIS and its associated invasive breast cancer tissues was performed to assess DNA methylation changes throughout TN-DCIS progression to canine TNBC. We have also used established DNA methylation patterns to identify changes specific to TN-DCIS that could potentially be used to predict TN-DCIS that will not progress to invasive TNBC.

## Results

### Overview of genome-wide changes in promoter DNA methylation during different stages of triple-negative DCIS progression

**Figure 1:**
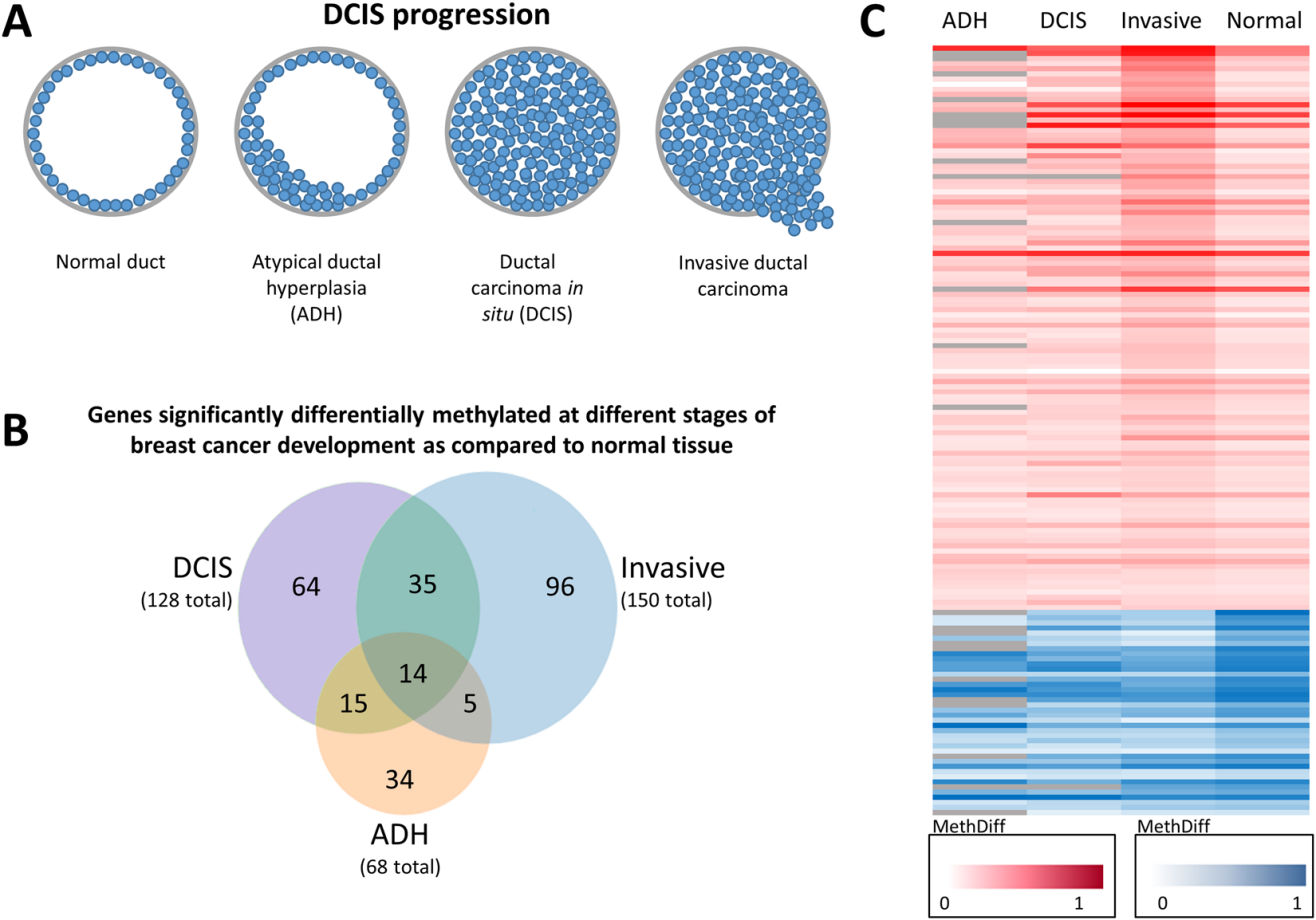
Global changes in promoter DNA methylation during stages of triple-negative DCIS progression. (**A**) Progressive stages of ductal carcinoma *in situ* (DCIS) to invasive breast cancer are shown. (**B**) Venn diagram representing distribution and overlap of significantly differentially methylated genes in ADH, DCIS, and invasive stages of TN-DCIS progression breast cancer. (**C**) The heat map showing differentially methylated genes in ADH, DCIS, and invasive breast cancer as compared to healthy tissue. Red represents genes that are hypermethylated, and blue indicates genes that are hypomethylated compared to healthy tissue.

Typically, milk ducts in the breast contain a monolayer of epithelial cells that proliferate and turnover at a controlled rate. The ADH stage is characterized by intraductal epithelial cell proliferation. A finding of ADH indicates breast cancer risk but is not considered precancerous or cancer. On the other hand, DCIS is classified as a precancerous stage and often is referred to as a non-invasive or pre-invasive lesion. In DCIS, cells that line the milk ducts proliferate out of control but are contained in the milk ducts and have not escaped to surrounding breast tissue or other distant tissues. Invasive breast cancer, however, known as invasive ductal carcinoma (IDC), is a tumor that started in the milk duct and has invaded tissues of the surrounding breast and potentially other distant sites^37^ (Fig. 1A). IDC makes up 80% of all histological types of breast cancer cases^38^. Epigenetic alterations, particularly changes in DNA methylation that occur throughout these stages, could distinguish stages and be potentially used to predict progression from ADH to DCIS and subsequently to IDC.

Following RRBS, we applied Illumina base-calling software to delineate DNA methylation patterns in healthy canine breast tissue, breast tissue from ADH, DCIS, and IDC. When comparing methylation status in canine ADH versus healthy breast tissue, 68 promoters were significantly differentially methylated (*P* < 0.05, Fisher’s exact test) (Fig. 1B), 38 of which were hypomethylated and the remaining 30 hypermethylated in ADH. Interestingly, among genes associated with hypomethylated promoters, there was a cancer promoting gene *PLAU*. According to gene ontology (GO) function and KEGG pathway analysis using DAVID Knowledgebase (Fig. S1), *PLAU* is categorized into “transcriptional misregulation,” the most prominent pathway of genes with hypomethylated promoters in ADH versus healthy tissue. This finding indicates that disruption of normal cell functions is present even in very early stages of progression to breast cancer.

Among 128 promoters significantly differentially methylated in the pre-invasive DCIS stage compared to healthy breast tissue (*P* < 0.05, Fisher’s exact test) (Fig. 1B), 80 were hypomethylated and 48 were hypermethylated. Hypomethylation predominates in this stage and corresponds to several pathways associated with immune and inflammatory response (Fig. S1). For example, *CCL1*, *TGFB1*, and *TLR9* play roles in immunoregulatory processes, activation of inflammatory pathways, and facilitating innate/adaptive immunity, respectively. Along the same line, hypermethylation of *IL-13*, an anti-inflammatory cytokine, could also contribute to the observed targeting of inflammation and immune regulation during the DCIS stage.

In contrast to earlier stages, hypermethylation dominates in the invasive breast cancer stage as opposed to hypomethylation, with 40 promoters hypomethylated and 110 promoters hypermethylated (*P* < 0.05, Fisher’s exact test) (Fig. 1B). Function and pathway analyses suggest that this widespread differential methylation leads to the disruption of many processes such as cell proliferation and migration, phosphorylation, focal adhesion, and TNF signaling pathway (Fig. S1). Several genes with hypermethylated promoters are established tumor suppressor genes such as *DLC1* and *CASP3*, while examples of hypomethylated genes with cancer-promoting functions include *MADCAM1* and *CXCR3*. Our overall analyses of differentially methylated genes associated with invasive breast cancer indicates extensive dysregulation of cancer-related genes whose altered promoter methylation may have important functional consequences contributing to the invasive cancer phenotype.

Furthermore, differentially methylated promoters in invasive breast cancer show either consistent, variable, or opposite patterns of aberrant DNA methylation in earlier stages of progression as presented in the heat map in Fig. 1C. The heat map depicts 150 significantly differentially methylated promoters identified to be hypo- or hypermethylated in the invasive stage compared to normal breast tissue. Methylation status of those 150 gene promoters during stages preceding invasive cancer are indicated under the ADH and DCIS columns. 96 gene promoters are significantly hypo- or hypermethylated exclusively in invasive cancer stage, while 35 other promoters are hypo- or hypermethylated in both DCIS and invasive stages (Fig. 1B,C). In addition, some gene promoters are differentially methylated throughout all stages of DCIS progression (ADH, DCIS and invasive stages), but the direction of the difference varies across the stages (Fig. 1C). Upon identifying differential patterns of DNA methylation across stages of TN-DCIS progression, we elaborated on the functional relevance of the genes associated with DNA methylation changes to categorize and identify candidates that may determine stages of TN-DCIS progression.

### Alterations of gene-specific promoter DNA methylation patterns may distinguish stages of TN-DCIS progression to invasive breast cancer

#### Genes with differentially methylated promoters in both DCIS pre-invasive stage and invasive breast cancer

**Figure 2:**
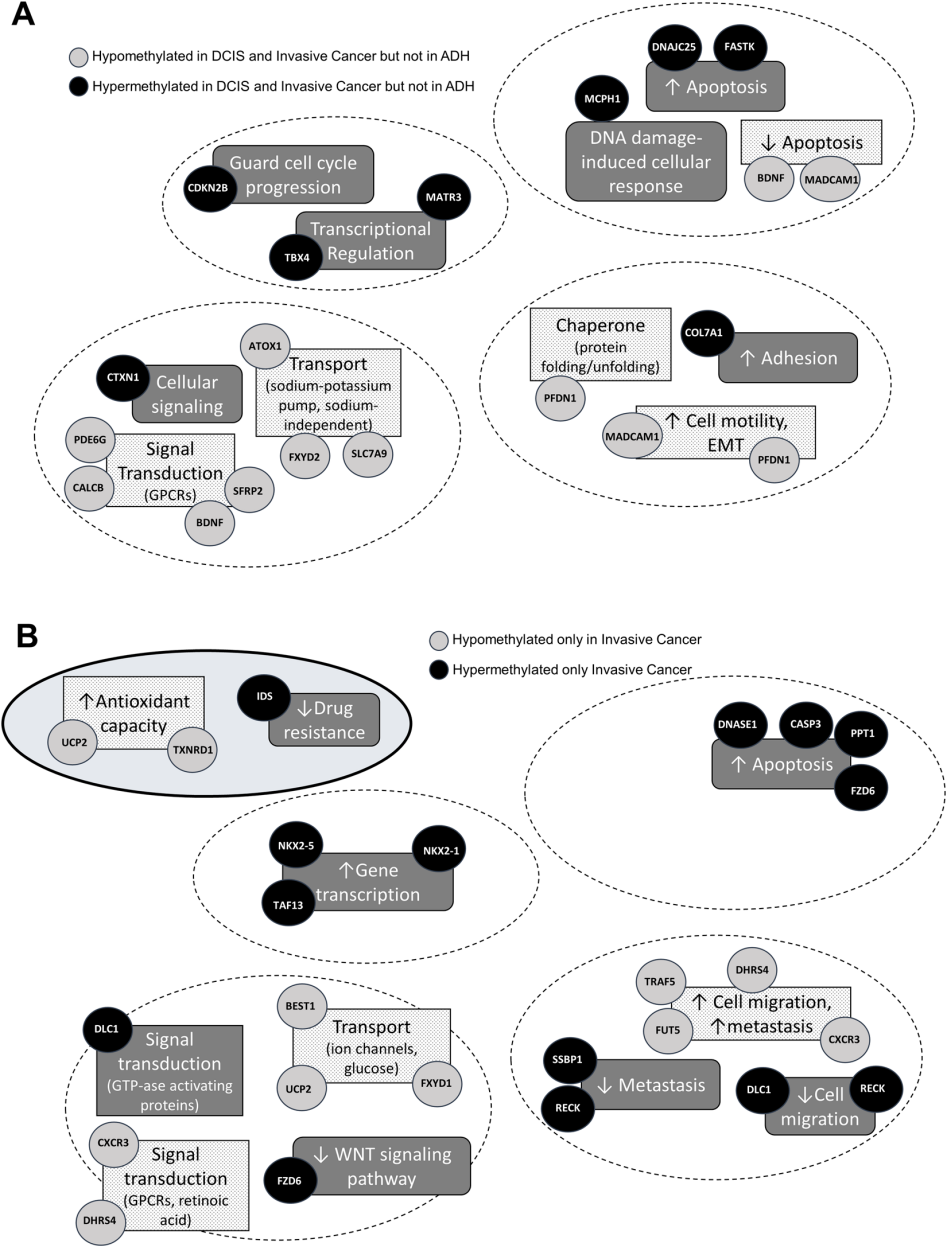
Promoter DNA methylation patterns are explicitly changed in the late stages of DCIS progression and invasive breast cancer. (**A**) Functional categories are containing genes that are hypomethylated and hypermethylated in DCIS and invasive stages but not in ADH breast cancer. Gray circles represent genes that are hypomethylated in DCIS and invasive stages. Black circles indicate genes that are hypermethylated in DCIS and invasive cancer. (**B**) Functional categories are containing genes that are hypomethylated and hypermethylated only in invasive breast cancer. Gray circles represent genes that are hypomethylated in invasive cancer. Black circles indicate genes that are hypermethylated in invasive cancer.

Functional analysis of genes differentially methylated in both DCIS pre-invasive stage and invasive breast cancer revealed several players involved in processes commonly dysregulated in cancer, such as cell cycle progression, transcriptional regulation, apoptosis and cellular signaling (Fig. 2A). We identified a gene that guards cell cycle progression, *CDKN2B*, to be hypermethylated and likely silenced in TN-DCIS and invasive stages of TNBC, potentially supporting uncontrolled cell proliferation characteristic of later stages of TN-DCIS progression. We found two genes that regulate transcription, *TBX4* and *MATR3*, to be hypermethylated. *TBX4* is a transcription factor that regulates genes involved in differentiation and reduced expression of this gene may be involved in the downregulation of histone demethylase pathways^39^. *MATR3* helps to stabilize mRNA species and silencing of this gene by DNA methylation could result in aberrant transcription of target genes^40^.

Genes regulating apoptosis, namely *DNAJC25*, *FASTK*, *BDNF*, and *MADCAM1*, were differentially methylated in TN-DCIS and invasive stages of TNBC. Hypermethylation of pro-apoptotic genes *DNAJC25* and *FASTK* in addition to hypomethylation of anti-apoptotic genes *BDNF* and *MADCAM1* could be responsible for the survival capability of cells at later stages of TN-DCIS progression. Hypermethylation of DNA damage-induced cellular response gene *MCPH1* could also provide cancer cells with a safeguard against cell death mechanisms. Several genes important in cellular signal transduction such as *PDE6G*, *CALCB*, *BDNF*, *SFRP2*, and transport such as *ATOX1*, *FXYD2*, *SLC7A9*, were hypomethylated in later stages of TN-DCIS progression, with the exception of *CTXN1*, a mediator of intracellular and extracellular signaling. The latter was the most highly hypermethylated gene in invasive breast cancer (DiffMeth = 0.43) (Table S1). Genes involved in transport such as *ATOX1*, a copper chaperone protein that functions as an antioxidant and is involved in breast cancer cell migration^41,42^ and *FXYD2*, a sodium/potassium-transporting ATPase subunit whose increased expression in tumors may contribute to angiogenesis^43^, were hypomethylated in TN-DCIS and invasive breast cancer. Hypomethylation of signal transduction-related genes *BDNF* and *CALCB* have the potential to lead to a myriad of cellular responses associated with cancer such as cell growth and proliferation, angiogenesis, and inflammation^44,45^. Genes involved in pathways related to increased cell motility, *PFDN1* and *MADCAM1*, were hypomethylated while a gene associated with increased cell adhesion, *COL7A1*, was hypermethylated in TN-DCIS and invasive stages of TNBC. Interestingly, *PFDN1*, an active oncogene that encodes for a chaperone protein essential for cytoskeletal assembly and whose overexpression is associated with epithelial-mesenchymal transition (EMT) and cell invasion, was the highest hypomethylated gene in both DCIS and in invasive cancer (DiffMeth = −0.67 and −0.65, respectively) (Table S1)^46^. Hence, DNA methylation alterations of these three genes may result in consequent expression changes and contribute to loss of adhesion and the process of EMT allowing the cells in later stages of DCIS progression to migrate and metastasize.

#### Genes with differentially methylated promoters specific to invasive breast cancer

Functional categories identified in the analysis of genes differentially methylated in late stages of TN-DCIS progression (DCIS and invasive stages) were also found to be associated with 96 genes differentially methylated exclusively in invasive cancer (Fig. 1B). Although functional categories were the same, different genes from those categories were specifically altered in the invasive cancer, indicating an additional layer of dysregulation occurring in this stage.

Factors that regulate gene transcription, such as *TAF13*, *NKX2–5*, and *NKX2-1*, pro-apoptotic genes, such as *DNASE1*, *CASP3*, *PPT1*, and *FZD6*, and several inhibitors of oncogenic signaling, migration and metastasis, namely *FZD6*, *RECK*, and *SSBP1*, were significantly hypermethylated explicitly in invasive breast cancer (Fig. 2B). *TAF13* has been shown to facilitate RNA polymerase II complex assembly and transcription initiation^47^. The inability to initiate transcription of essential tumor suppressor genes, due to hypermethylation and potential silencing of *TAF13*, could contribute to the aggressive phenotype characteristic of TNBC. *NKX2-1* is a transcription factor that regulates thyroid-specific genes and genes involved in morphogenesis. *NKX2-1* has previously been identified as a marker to distinguish breast cancer from other types of cancer^48^. *DNASE1*, *CASP3* and *PPT1* are involved in cell death by apoptosis^49–51^. Silencing of genes integral to pro-apoptotic mechanisms provides invasive cancer cells with the capacity to live and thrive in environments that typically promote programmed cell death. Negative regulator of Wnt oncogenic signaling, Frizzled receptor 6 (*FZD6*) is also hypermethylated in invasive breast cancer. This gene was shown to repress Wnt ligand-induced canonical signaling and reduce activation of β-catenin target genes^52^, leading to inhibition of Wnt-regulated cell proliferation. *DLC1* encodes for an RHO GTPase accelerating protein (GAP) that plays a role in the regulation of GTP binding proteins and functions as a potent tumor suppressor gene in several cancers including breast cancer^53,54^. *RECK*, a gene involved in suppressing cancer cell migration, invasion, and metastasis, negatively regulates matrix metalloproteinase 9 (MMP9) by directly inhibiting its enzymatic activity and abolishing MMP9 secretion^55^. In previous studies, regulation of *RECK* by DNA methylation has been described and proposed as a potential marker for predicting breast cancer prognosis^56^. Finally, mitochondrial single-stranded DNA binding protein (*SSBP1*) is a negative regulator of metastasis whose downregulation leads to reduced mitochondrial DNA copy number and consequent activation of TGFβ-induced EMT^57^. Hypermethylation and silencing of genes involved in processes necessary to control cell migration and invasion likely have a profound impact on the severe phenotype observed in invasive TNBC.

We also found that there are gene promoters specifically hypomethylated at the invasive breast cancer stage. Genes associated with signal transduction, such as *CXCR3* and *DHRS4*, and transport, such as *BEST1*, *UCP2*, and *FXYD1*, as well as increased cell migration and metastasis, namely *TRAF5*, *FUT5*, and *DHRS4*, were hypomethylated (Fig. 2B). *CXCR3*, an oncogene previously established as a prognostic marker for solid tumors, is a chemokine receptor involved in leukocyte trafficking and migration in response to chemotactic signals leading to tumor immunity and the promotion of cell migration and invasion^58^. *DHRS4* is an alcohol dehydrogenase within the *DHRS4* gene cluster that is regulated by a long-noncoding RNA called AS1DHRS4. Studies have shown that AS1DHRS4 recruits epigenetic machinery, including DNA methyltransferases (DNMTs) and histone modifiers, to the *DHRS4* gene cluster to regulate expression of *DHRS4*. A decrease in AS1DHRS4 has been associated with increased metastatic capacity in clear cell renal carcinoma via lesser recruitment of DNMTs to the *DHRS4* gene cluster^59^. The same phenomenon may be occurring in our breast cancer model leading to the hypomethylated state of the *DHRS4* promoter. *BEST1*, *UCP2* and *FXYD1* are genes associated with nutrient and energy transport that have previously been identified as upregulated in cancer cells^60–62^. *BEST1* and *UCP2* encode for transport proteins involved in ligand-gated chloride channels and proton leakage during oxidative phosphorylation, respectively^63,64^, whereas *FXYD1* is a subunit of sodium/potassium ATPase, important for active transfer through cell membranes. As for genes involved in signaling pathways whose alterations have the potential to disrupt a wide variety of cell processes, *TRAF5* and *FUT5* are two candidates that could have a robust impact. *TRAF5* links the tumor necrosis factor (TNF) family of proteins with other signal transduction pathways such as *NFκB*, *MAPK*, and *JNK*. *FUT5* is a gene involved in the fucosylation of glycans in circulating tumor cells leading to the initiation of tumor extravasation. Inhibition of fucosylation has been shown to reduce oncogenic properties of breast cancer; therefore, hypomethylation and subsequent activation of *FUT5* to increase fucosylation could exacerbate oncogenic properties^65^.

Most importantly, a functional category that was specific to invasive breast cancer was the increased antioxidant capacity of cells. Specifically, *UCP2* and *TXNRD1*, key players in oxidative stress and redox homeostasis, were hypomethylated^66,67^. Hypomethylation and upregulation of these antioxidant-related genes could suggest that cancer cells can deal with oxidative stress and even take advantage of antioxidant-related mechanisms to ensure their survival. It also appears that invasive breast cancer gains function that contributes to drug resistance. For instance, hypermethylation and potentially reduced expression of *IDS*, a sulphatase whose silencing may lead to accumulation and export of estrogen sulfates contributing to multidrug resistance, could play a role in the problematic nature of treating TNBC^68^.

#### Genes with differentially methylated promoters specifically in DCIS pre-invasive stage

**Figure 3:**
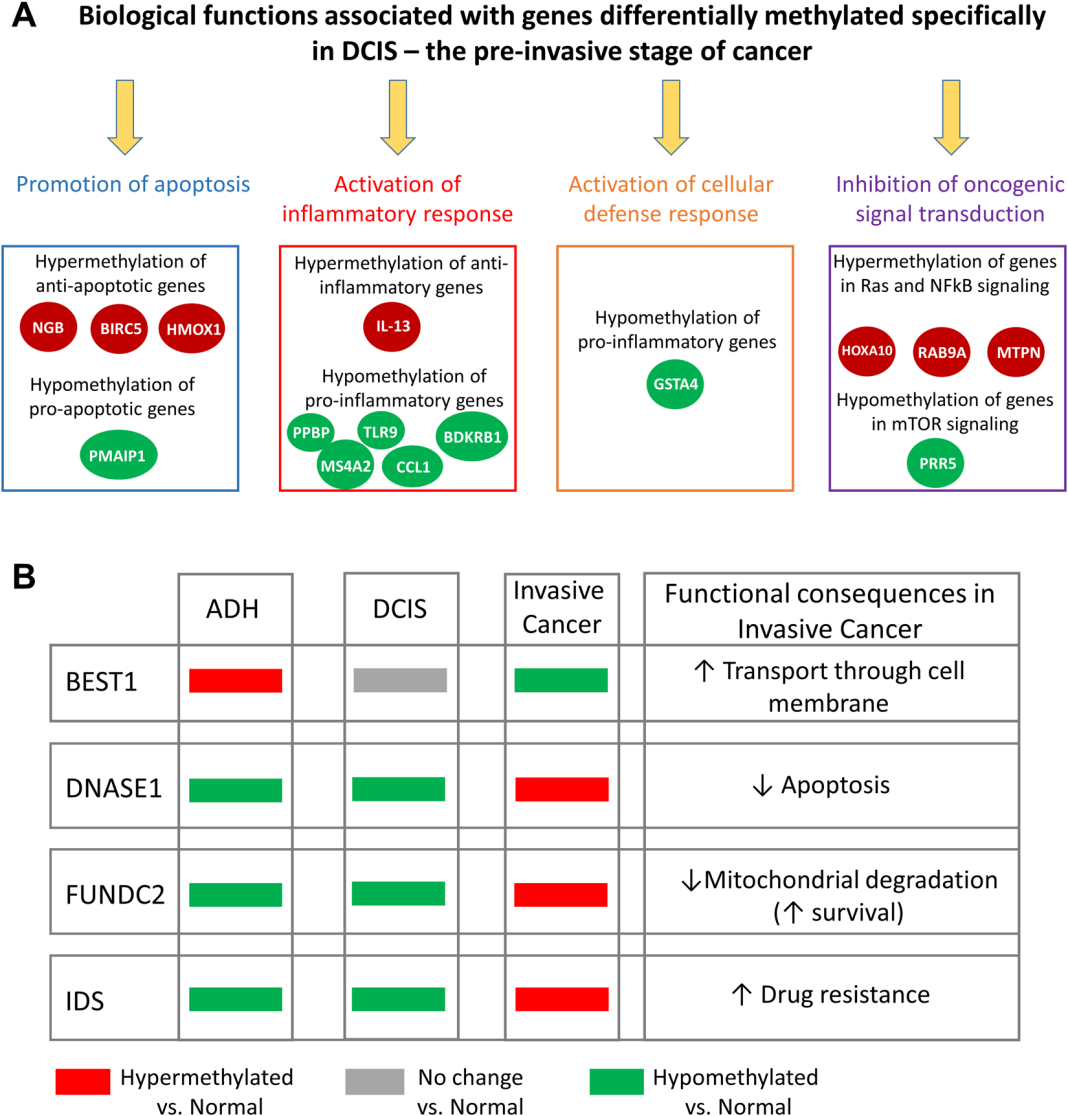
Promoter DNA methylation patterns can be used as DCIS markers. (**A**) Biological functions of genes differentially methylated in the pre-invasive DCIS stage of breast cancer are shown. Red circles represent genes that are hypermethylated and green circles show genes that are hypomethylated in the DCIS stage as compared to healthy breast tissue. (**B**) The methylation status of selected genes in ADH, DCIS, and invasive breast cancer is shown. *BEST1*, *DNASE1*, *FUNDC2*, and *IDS* genes were found to show the opposite methylation trend in earlier (ADH and DCIS) stages compared to invasive stages of breast cancer.

Differentially methylated genes specific to DCIS fall into functional categories that highlight the capacity for DCIS cells to fight progression to invasive cancer and uphold cellular processes associated with the non-invasive and non-cancerous phenotype. Functions such as the promotion of apoptosis, activation of inflammatory and immune responses, and inhibition of oncogenic signaling are maintained and active during the DCIS stage (Fig. 3A). For example, *NGB* encodes for an anti-apoptotic neuroglobin protein which acts as a sensor of oxidative stress, hypoxia, and nutrient deprivation in breast cancer cells^69,70^. In our model of TN-DCIS progression, *NGB* is hypermethylated only in the DCIS pre-invasive stage. Hypermethylation and potential silencing of this anti-apoptotic stress sensor would disallow breast cancer cell adaptation and shift the balance toward promoting programmed cell death in response to the changing cellular microenvironment.

Additionally, *BIRC5*, a negative regulator of apoptosis through promoting cell proliferation and suppressing cell death^71^, is specifically hypermethylated in the DCIS stage. Silencing of this gene could contribute to the upkeep of standard cell death mechanisms in the DCIS stage. Cell death pathways may be further strengthened by hypomethylation-activated *PMAIP1*, a gene that promotes activation of caspases involved in apoptosis.

Activation of inflammatory and immune response is another functional category associated with genes differentially methylated only in the DCIS stage. Several genes from cytokine and chemokine signaling are significantly differentially methylated in the pre-invasive stage of TN-DCIS progression but not in invasive breast cancer. *IL-13* is an anti-inflammatory cytokine that downregulates macrophage activity, thereby inhibiting the pro-inflammatory cascade. In our model, *IL-13* is hypermethylated in the DCIS stage only, which potentially increases the activity of cells to fight inflammation. Hypomethylation and potential activation of *PPBP*, *TLR9*, *MS4A2*, *CCL1*, and *BDKRB1* may allow innate immune cells to target sites of inflammation and appropriately counteract inflammation-inducing processes. *TLR9* is a toll-like receptor responsible for controlling the pathogen-induced immune response. *PPBP* is a platelet-derived growth factor and chemokine ligand that acts as a chemoattractant and activator of neutrophils to induce DNA synthesis, glycolysis, and secretion of inflammation mediators. *CCL1* is also a chemokine ligand but is a chemoattractant of monocytes instead of neutrophils. Taken together, the upregulation of *PPBP* and *CCL1* likely aids in the trafficking of immune cells.

Furthermore, genes involved in cellular defense and inhibition of oncogenic signaling are differentially methylated in the DCIS stage only. For example, hypomethylated *GSTA4* encodes for an enzyme involved in defense against toxic and carcinogenic compounds. Hypomethylation of this gene promoter could lead to its upregulation and appropriate cellular response to unwanted invaders in the changing environment. Hypermethylated *RAB9A* is involved in GTPase-mediated signal transduction and protein transport between endosomes and the Golgi network. *RAB9A* has been shown to be upregulated in an aggressive subpopulation of cells associated with metastatic breast cancer phenotype^72^. Therefore, *RAB9A* hypermethylation and potential downregulation could be a contributing factor to maintaining the DCIS stage without progression to invasive breast cancer. An early study defining differentially expressed genes distinguishing DCIS from IDC identified *MTPN* as one of the most frequently differentially expressed genes between DCIS and IDC^73^. MTPN promotes dimerization and activity of NFκB, leading to the promotion of cell growth. Interestingly, *MTPN* is hypermethylated in DCIS in our study, which could conseqently suppress uncontrolled cell growth and disallow the advancement to invasive breast cancer. Hypermethylated *PRR5*, an established tumor suppressor gene in breast cancer, is a component of mTOR complex 2 which acts as a central regulator of cell growth and survival in response to hormonal signals.

DNA methylation and potential expression alterations of genes in the aforementioned pathways indicate that mammary tissue in the DCIS stage maintains the capacity to promote apoptosis, activate inflammatory and immune responses, and disable oncogenic signaling. Because these alterations in DNA methylation do not seem to persist in the invasive stage of TNBC in our model, we propose that they can be evaluated in further studies as indicators of the DCIS stage and provide guidance for determining whether DCIS would progress to invasive breast cancer.

#### Genes with opposite direction of differential DNA methylation in different stages of TNBC progression

A subset of 4 genes show altered patterns of DNA methylation in ADH, DCIS, and invasive stages compared to healthy breast tissue in our canine model, however the direction of DNA methylation change varies between the stages (Fig. 3B). While *BEST1* is hypomethylated in invasive breast cancer compared to healthy breast tissue, it is hypermethylated in ADH and without any change in DCIS stage. *BEST1* is involved in the transport of ions through the cell membrane by forming calcium-activated chloride ion channels in epithelial cells^63^. The upregulation of *BEST1* in colon cancer cells has been shown to significantly increase cell growth, indicating *BEST1* as an essential accelerator of cell proliferation^60^. A switch from hypermethylation in hyperplasia to hypomethylation and potential activation of *BEST1* in invasive cancer could have a notable impact on nutrient uptake and cellular response, allowing breast cancer cells heightened capacity to grow and proliferate.

*DNASE1*, *FUNDC2*, and *IDS* are genes hypermethylated in invasive breast cancer stage, although they demonstrate detectable hypomethylation in ADH and DCIS stages. Taking into account the functions of these genes, their hypomethylation and potential activation may aid to promote cell death by apoptosis (*DNASE1*)^50^, induce degradation and turnover of mitochondria to facilitate necessary cell turnover (*FUNDC2*), and convert estrogen sulfates into free estrogens to protect from breast cancer resistance protein (BCRP)-mediated drug resistance (*IDS*)^68^ during ADH and DCIS stages. On the other hand, hypermethylation of *DNASE1*, *FUNDC2*, and *IDS* during the invasive stage may result in cells’ capablility to relieve themselves of safeguards from programmed cell death and cell turnover. The switch of DNA methylation status in invasive breast cancer stage makes these 4 gene candidates potentially useful prognostic markers in distinguishing progression from DCIS to invasive breast cancer.

### Differential DNA methylation of gene candidates associated with canine TN-DCIS progression is functionally linked to gene transcriptional activity

**Figure 4:**
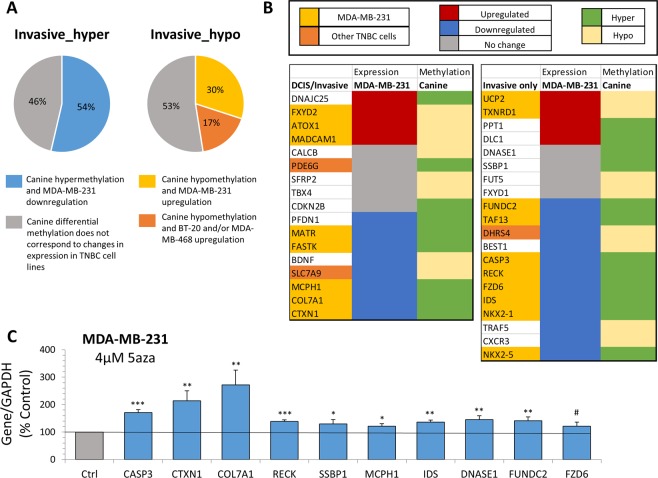
Analysis of expression of canine gene candidates in human triple negative breast cancer cell lines. (**A**) Pie charts depict percentage of concordance (shown in color) between canine methylation status and human breast cancer cell expression patterns. Grey portion represents lack of corresponding changes between DNA methylation and gene expression, or no change in gene expression. Left panel represents 110 significantly hypermethylated gene promoters in invasive stage of canine breast cancer. Right panel represents 40 significantly hypomethylated gene promoters in invasive stage of canine breast cancer. Each set of differentially methylated gene promoters in canine samples was compared to expression levels in TNBC cell lines (MDA-MB-231, BT-20, and MDA-MB-468). (**B**) Heat maps of gene expression in MDA-MB-231 and methylation status associated with candidate genes in canine samples. Left panel represents all canine candidates differentially methylated in DCIS and invasive stages. Right panel represents all canine candidates differentially methylated only in invasive stage of DCIS progression. Gene names highlighted in yellow show genes where changes in DNA methylation in canine samples correspond to changes in gene expression in MDA-MB-231 cells. Gene names highlighted in orange show genes where changes in DNA methylation in canine samples correspond to changes in gene expression in the other TNBC cell lines analyzed (BT-20 and MDA-MB-468) (**C**) Gene expression for hypermethylated candidates in canine samples upon 72 hour treatment of MDA-MB-231 triple negative breast cancer cells with 4 μM 5-aza as determined by qPCR. All experiments represent mean ± SD of three independent experiments; ***P < 0.001, **P < 0.01, *P < 0.05, ^#^P < 0.10.

In order to provide a functional link between changes in DNA methylation and gene expression and relate the findings in the canine breast cancer samples with human breast cancer, we have utilized publicly available RNA sequencing data of ER-negative human breast cancer cell lines from a study by Sun and collegues (GEO accession number: GSE27003)^74^. Gene expression signatures of ER-negative MDA-MB-231, BT-20 and MDA-MB-468 breast cancer cells as compared with mammary epithelial MCF10A cells were established^74^. Methylation status of all genes with differentially methylated promoters associated with invasive TNBC in our canine model was compared to gene expression in human MDA-MB-231, BT-20 and MDA-MB-468 breast cancer cells to assess whether differential DNA methylation is linked to corresponding changes in gene expression. We found that among the genes associated with hypermethylated promoters, 59 genes (54%) were downregulated as expected. Among genes associated with hypomethylated promoters, 12 genes were upregulated in MDA-MB-231 cells (30%) and increased expression for additional 7 genes was also observed in BT-20 and MDA-MB-468 cell lines (17%) (Fig. 4A).

Differentially methylated gene candidates that distinguish TN-DCIS from invasive TNBC in our canine model were specifically examined. We found that among the 17 candidates associated with DCIS and invasive stages of DCIS progression, 10 genes displayed corresponding changes in expression patterns. For example, the increased expression of *ATOX1* and *MADCAM1* in MDA-MB-231 breast cancer cells corresponded with their promoter hypomethylation in canine DCIS and invasive cancer samples. Some of the most robustly downregulated gene candidates such as *CTXN1*, *COL7A1*, and *MCPH1* were among hypermethylated candidates distinguishing the DCIS and invasive stages in canine TN-DCIS progression. Additionally, for 11 out 20 differentially methylated gene candidates that discriminate invasive stage of TNBC from earlier pre-invasive stages, corresponding changes in gene expression were detected in TNBC cell lines. In fact, several tumor suppressor genes such as *CASP3*, *RECK*, *FZD6*, and *NKX2* were strongly downregulated in MDA-MB-231 breast cancer cells and exhibited promoter hypermethylation in the canine TNBC samples (Fig. 4B). Of note, several differentially methylated canine gene candidates exhibited no change in human gene expression (Fig. 4B, respresented in grey). Some genes with no change in expression had very low RNA sequencing read counts in the human TNBC cell line data. Deeper RNA sequencing could be required to reveal additional changes in gene expression.

Higher concordance between DNA methylation in our canine model and gene expression in human TNBC cell lines, especially MDA-MB-231 cells, was detected for genes hypermethylated in invasive cancer compared to normal tissue (54% alignment). Therefore, we sought to further investigate the regulatory role of DNA methylation related to hypermethylated canine gene candidates in MDA-MB-231 breast cancer cells. We treated MDA-MB-231 breast cancer cells with a demethylating drug and potent DNA methyltransferase inhibitor, 5-aza-2′-deoxycytidine (5-aza), at 4 μM concentration for 72 hours. Gene expression in response to 5-aza treatment was measured using qPCR for 10 candidate genes found to be hypermethylated in DCIS and/or invasive stages of the TN-DCIS canine model. Compared to vehicle-treated cells (PBS), significant upregulation of 9 of the 10 canine candidate genes was detected in 5-aza-treated cells, with the remaining gene demonstrating a trend for upregulation (P = 0.08) (Fig. 4C). All 10 of the tested genes were described to have tumor suppressive functions. The upregulation of *CASP3*, *CTXN1* and *COL7A1* upon 5-aza treatment was most robust with 70%, 114%, and 172% increased expression, respectively. The other gene candidates showed significant upregulation of gene expression by 30–45% compared to vehicle-treated cells (Fig. 4C). These findings indicate that DNA methylation at least partially controls expression of these candidate genes in human breast cancer cells, and upon inhibiting DNA methyltransferase activity these genes are reactivated.

### Gene candidates differentially methylated in stages of canine TN-DCIS progression demonstrate altered expression patterns in human clinical samples of DCIS and invasive breast cancer

**Figure 5:**
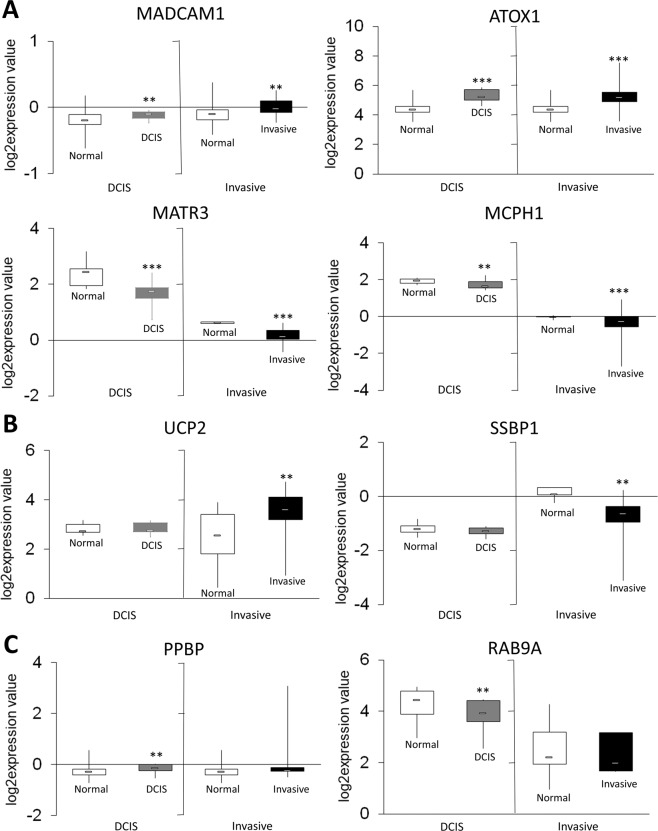
Gene candidates from canine analysis of TN-DCIS progression have altered expression in human DCIS and invasive breast cancer. (**A**) Graphs show expression levels of *MADCAM1, ATOX1, MATR3*, and *MCPH1* genes in normal, DCIS, and invasive stages of breast cancer. *MADCAM1 and ATOX1* show significant over-expression in DCIS and invasive cancer as compared to normal tissue. *MATR3* and *MCPH1* are significantly under-expressed in DCIS and invasive cancer. (**B**) Expression levels of *UCP2* and *SSBP1* in normal, DCIS, and invasive breast cancer are shown. *UCP2* is significantly over-expressed in invasive cancer compared to normal tissue. *SSBP1* is downregulated significantly in invasive cancer compared to normal breast tissue. (**C**) Expression levels of *PPBP* and *RAB9A* in normal breast tissue, DCIS, and invasive cancer are shown. *PPBP* is significantly upregulated, and *RAB9A* is significantly downregulated in the DCIS stage only. All data obtained from the Oncomine publically available database. *p < 0.05, **p < 0.01 and ***p < 0.001.

Next, we used publicly available datasets from Oncomine and compared differential DNA methylation in the canine model to expression in human clinical samples. Oncomine provides human gene expression data from many clinical studies and multiple stages of cancer progression. In Fig. 5, we present gene expression levels of select gene candidates that: (1) distinguish later stages of DCIS progression (DCIS and invasive) from ADH or healthy breast tissue, (2) distinguish invasive breast tissue from preceding stages (DCIS, ADH, and healthy breast tissue), and (3) distinguish DCIS from all other stages. Each boxplot represents gene expression data from the DCIS stage (grey) and invasive ductal carcinoma (black) compared to healthy breast tissue (white). Gene candidates hypomethylated (*MADCAM1* and *ATOX1*) and hypermethylated (*MATR3* and *MCPH1*) in DCIS and invasive stages of canine TNBC progression show corresponding changes in gene expression in human breast tissues (Fig. 5A). Genes specifically differentially methylated in invasive breast cancer, *UCP2* and *SSBP1*, show no change in gene expression in DCIS but significant change in gene expression in invasive breast cancer tissue from human clinical samples (Fig. 5B). This observation emphasizes the importance of these gene candidates in marking the invasive stage and further suggests that expression could be an additional parameter for distinguishing the stages. The correlation between methylation and expression for DCIS-specific markers further supports this suggestion. As shown in (Fig. 5C), *PPBP* and *RAB9A*, show a significant change in gene expression in DCIS but no change in gene expression in invasive breast cancer tissue from human clinical samples.

## Discussion

Triple-negative breast cancer is an invasive and aggressive subtype of breast cancer that has proven difficult to treat due to the lack of effective therapies. Some successful ways of treating other breast cancer subtypes have been discovered, such as endocrine therapy targeting hormone receptors (ER and PR) or therapies targeting HER2, but because of the lack of receptors in triple-negative breast cancer subtype (ER-, PR-, and HER2-negative), these therapies do not benefit TNBC patients. Thus, patients with TNBC require specialized treatment approaches^75,76^. In addition, standard breast cancer screening methods like mammography cannot determine the magnitude of severity or molecular characteristics of breast tumors (i.e., TNBC status) resulting in a need for additional molecular testing. Such testing to date has not yet been extensively developed despite the fact that TNBC makes up about 15%-20% of breast cancer cases and results in a disproportionate number of breast cancer deaths^3^. Therefore, the concept of developing accurate ways to detect TNBC before it becomes invasive has been proposed. DCIS is a pre-invasive stage characterized by increased growth and proliferation of cells that line the milk duct that precedes IDC. Although not considered cancer, DCIS accounts for almost one-third of breast cancers diagnosed by mammography. Furthermore, longitudinal studies following patients diagnosed with DCIS revealed that only 20–50% of DCIS progressed to invasive breast cancer after 30 year follow-up^77^. These statistics indicate the need for establishing methods that will predict which of the DCIS cases will progress to invasive breast cancer and which cases will not.

Another pressing issue is the over-diagnosis of breast cancer. Systematic reviews from 2007 and 2016 reported breast cancer over-diagnosis estimates ranging from 0 to over 50%^78,79^. Years later, over-diagnosis is still considered the most severe downside of breast cancer screening^80^. The heterogeneous nature of breast cancer makes it difficult to determine from existing screening methods whether or not breast tumors would proliferate and metastasize, grow and develop at a slow pace, or not progress at all.

Our study establishes gene candidates exhibiting differential promoter DNA methylation throughout stages of TN-DCIS progression, from normal breast tissue to early-stage hyperplasia (ADH), pre-invasive stage (DCIS), and further to invasive breast cancer (Fig. 1). For the first time, we use a canine model of triple-negative invasive breast cancer to follow all stages of progression of TN-DCIS to invasive breast cancer and identify DNA methylation alterations to distinguish changes that may predict progression. We determined significantly differentially methylated genes common in DCIS and invasive stages of TN-DCIS progression (Fig. 2A) that could be used to delineate late stages. We further discovered genes specific to the invasive breast cancer phenotype (Fig. 2B) whose DNA methylation changes only when breast cancer has progressed to IDC, and genes that are differentially methylated only in DCIS and not in normal breast tissue, ADH or invasive cancer (Fig. 3A). The latter two groups of genes could potentially be used as indicators of which cases of DCIS may progress to invasive breast cancer and which are less likely to progress. We also identified a particularly interesting subset of gene promoters where the direction of change in DNA methylation differs drastically between invasive stage and earlier stages (Fig. 3B). These genes may be the most functionally relevant set of candidates due to their opposite pattern of differential DNA methylation in invasive stage. In order to strengthen this point, we analyzed RNA sequencing data from human TNBC cell lines to understand whether changes in DNA methylation in canine samples correspond with functional gene expression changes. We found a substantial amount of agreement between DNA methylation status of canine gene candidates and gene expression patterns in human breast cancer cell lines (Fig. 4). Further, we experimentally tested in human TNBC cells the role of DNA methylation in regulating expression of several canine gene candidates using a DNMT inhibitor, 5-aza. We discovered robust reactivation of several hypermethylated canine gene candidates in triple negative MDA-MB-231 breast cancer cells upon DNA methylation inhibition (Fig. 4), suggesting that transcriptional activity of these genes is at least partially regulated by the status of DNA methylation within promoter regions.

A 2009 National Institutes of Health (NIH) State-of-the-Science conference concluded that more focus should be made on accurate identification of patient subsets diagnosed with DCIS to stratify patients who can be managed with less therapeutic intervention and those who may be at higher risk of progressing to invasive breast cancer^81^. Indeed, research to identify molecular markers of DCIS and invasive breast cancer has surfaced in recent years. Several groups have sought to establish DNA methylation alterations as markers of breast cancer diagnosis and progression. Many of these studies have used human breast cancer tissues to identify changes in DNA methylation at selected regions^82,83^, while others have used human tissues to find candidates in a more exploratory manner (i.e., microarrays)^84,85^. Studies compare DNA methylation status of normal-adjacent breast tissues to multiple stages of progression to invasive breast cancer (i.e., hyperplasia, DCIS)^83^, only compare DCIS and invasive breast cancer versus normal-adjacent tissues^84,86^, only compare normal-adjacent breast tissue to invasive breast cancer^82^ or compare metastatic versus non-metastatic breast cancer^85^. Currently published studies evaluating DCIS progression to invasive breast cancer do not define DCIS progression in the triple-negative context, nor do they capture stages from healthy to invasive through ADH and DCIS in one model.

In our study of canine TN-DCIS progression to invasive TNBC, we define DNA methylation patterns associated with cancer-related genes to be altered across various stages of progression. We propose changes in many genes involved in a variety of functional categories to be indicators of late-stage (DCIS and invasive), invasive stage only or DCIS stage only. We also provide evidence for functional role of DNA methylation in regulation of expression of the established candidates that differentiate stages of progression of hyperplastic lesions to DCIS and TNBC. Once verified in other models and in human populations, the DNA methylation alterations reported in our study have the potential to be utilized in clinics to distinguish TN-DCIS progression stages.

## Materials And Methods

### Clinical specimens

All animal work was conducted in accordance with a protocol approved by the Purdue Animal Care and Use Committee (PACUC) and all animal procedures were carried out following the PACUC guidelines and overseen by the Laboratory Animal Program (LAP) at Purdue University. For this pilot study, progressing tissues from atypical ductal hyperplasia (ADH), ductal carcinoma *in situ* (DCIS), and invasive cancer and adjacent healthy tissues were collected from the same mammary gland from the same dog (n = 3). Mammary tissues were then formalin-fixed, paraffin-embedded, and reviewed by a board-certified pathologist to confirm the diagnosis and define lesions for dissection. Samples were immunohistochemically stained for ER, PR, and HER-2 expression.

### DNA extraction

Healthy, ADH, DCIS, and invasive carcinoma cells (cellularity > 90%) were collected from serial 8- to 10-μm thick paraffin slides using a scalpel or laser microdissected (Arcturus® LCM) to isolate areas of interest from the surrounding tissue. DNA was recovered using RecoverAll Total Nucleic Acid Isolation Kit by Ambion (Life Technologies, Carlsbad, CA). Briefly, 50 or 100 μl digestion buffer was added to each sample (consisting of 10 mM TRIS–HCl pH 8.3, 0.5% Tween 0.20, 1 mM EDTA) and 10 or 20 μl proteinase K (10 mg/ml, Roche, Almere, The Netherlands) was added and heated in a 56 °C water bath for 16 h. The genomic DNA was then extracted following the manufacturer’s instructions and quantified using a NanoDrop spectrophotometer.

### Reduced representation bisulfite sequencing (RRBS)

#### EpiQuest library construction

DNA samples were shipped on ice to Zymo Research (Irvin, CA) for EpiQuest library preparation and genome-wide DNA methylation analysis by reduced representation bisulfite sequencing (RRBS). Briefly, 200–500 ng of genomic DNA were digested first with TaqI followed by digestion with MspI (Ipswich, MA, USA). Size-selected TaqI-MspI fragments (40–120 bp and 120–350 bp) were filled-in and 3′-terminal-A extended, then extracted by Zymo Research DNA Clean and Concentrator-5 kit (Irvin, CA). Ligation to pre-annealed adapters containing 5′-methyl-cytosine was performed using Illumina’s DNA preparation kit and protocol (San Diego, CA). Purified adaptor-ligated fragments were bisulfite-treated using the EZ DNA Methylation-Direct Kit (Irvin, CA). Preparative-scale PCR was performed. DNA Clean and Concentrator-purified PCR products were subjected to a final size selection on a 4% NuSieve 3:1 agarose gel. SYBR green-stained gel slices containing adaptor-ligated fragments of 130–210 bp or 210–460 bp in size were excised. Library material was recovered from the gel (Zymoclean Gel DNARecovery Kit, Irvin, CA, USA) and sequenced on an Illumina HiSeq Genome Analyzer (San Diego, CA).

#### Sequence alignments and data analysis

Sequence reads from bisulfite-treated EpiQuest libraries were identified using standard Illumina base-calling software and then were analyzed using a Zymo Research proprietary analysis pipeline according to the manufacturer’s recommendations (Zymo Research, CA, USA). Residual cytosines in each read were first converted to thymines, with each such conversion noted for subsequent analysis. A reference sequence database was constructed from the 50 bp ends of each computationally predicted MspI-TaqI fragment in the 40–350 bp size range. All cytosines (Cs) in each fragment were then converted to thymines (Ts); the converted reads were aligned to the converted reference. The number of mismatches in the induced alignment was counted between the unconverted read and reference, ignoring cases in which a T in the unconverted read matched to a C in the unconverted reference. For a given read, the best alignment was kept if the second-best alignment had 2 more mismatches; otherwise, the read was discarded as non-unique. The methylation level of each sampled cytosine was estimated as the number of reads reporting a C divided by the total number of reads reporting a C or T. Fisher’s exact test or *t*-test was used for each CpG site that has at least 5 reads covered. Also, promoter, gene body, and CpG island annotations were added for each CpG. The software pipeline is implemented in Python.

### Cell culture and 5-aza-2′-deoxycytidine (5-aza) treatment

Human triple negative breast cancer MDA-MB-231 cell line was cultured in Dulbecco’s modified eagle medium (Gibco) supplemented with 10% fetal bovine serum (Gibco), 1U/ml penicillin and 1 µg/ml streptomycin (Gibco). Cells, grown in a humidified atmosphere of 5% carbon dioxide at 37 °C, were treated with 5-aza-2′-deoxycytidine (5-aza, Sigma-Aldrich) freshly resuspended in PBS. 24 h prior to treatment, cells were plated at a density of 3 × 10^5^ followed by exposure to 5-aza at 4 µM concentration for 72 hours. 4 µM concentration of 5-aza for 72 hours was determined in our previous studies to be the IC50 concentration^87^.

### RNA isolation and qPCR

TRIzol (Invitrogen) was used to isolate total RNA which served as a template for cDNA synthesis with AMV reverse transcriptase (Roche Diagnostics), according to the manufacturer’s protocol. Amplification reaction was performed in CFX96 Touch Real-Time PCR Detection System (Bio-Rad) using 2 µl of cDNA, 400 nM forward and reverse primers (please see Supplementary Table S2 for sequences), and 10 µl of SsoFast EvaGreen Supermix (Bio-Rad) in a final volume of 20 µl. The following cycles were used in the amplification reaction: denaturation at 95 °C for 10 min, amplification for 60 cycles at 95 °C for 10 s, annealing temperature for 10 s, 72 °C for 10 s, and final extension at 72 °C for 10 min. The CFX Maestro Software (Bio-Rad) was used to quantify gene expression with a standard curve-based analysis.

## Supplementary information

Supplementary Material.
