# Author Correction: DNA methylation landscape of triple-negative ductal carcinoma *in situ* (DCIS) progressing to the invasive stage in canine breast cancer

**DOI:** 10.1038/s41598-020-62220-7

**Published:** 2020-03-19

**Authors:** Megan Beetch, Sadaf Harandi-Zadeh, Tony Yang, Cayla Boycott, Yihang Chen, Barbara Stefanska, Sulma I. Mohammed

**Affiliations:** 1 Food, Nutrition & Health Program, Faculty of Land and Food Systems, University of British Columbia, Vancouver, Canada; 2 Department of Comparative Pathobiology, Purdue University, West Lafayette, IN USA; 3 Purdue University Center for Cancer Research, Purdue University, West Lafayette, IN USA

Correction to: *Scientific Reports* 10.1038/s41598-020-59260-4, published online 12 February 2020

The original version of this Article contained a typographical error in the spelling of the author Sulma I. Mohammed, which was incorrectly given as Sulma Mohammed. This has now been corrected in the PDF and HTML versions of the Article, and in the accompanying Supplementary Information file.

