# Supplementary material for: DNA methylation landscape of triple-negative ductal carcinoma in situ (DCIS) progressing to the invasive stage in canine breast cancer
Source: Sci Rep. 2020 Feb 12;10:2415. doi: 10.1038/s41598-020-59260-4 (PMC7015930; doi:10.1038/s41598-020-59260-4)
Supplement: Supplementary file 1 — Supplementary Material. [file 41598_2020_59260_MOESM1_ESM.pdf]

Supplementary Information:

DNA methylation landscape of triple-negative ductal carcinoma *in situ* (DCIS) progressing to the invasive stage in canine breast cancer

Megan Beetch[1], Sadaf Harandi-Zadeh[1], Tony Yang[1], Cayla Boycott[1], Yihang Chen[1], Barbara Stefanska[1*], and Sulma Mohammed[2,3*]

[1]Food, Nutrition & Health Program, Faculty of Land and Food Systems, University of British Columbia, Vancouver, Canada

[2]Department of Comparative Pathobiology, Purdue University, West Lafayette, IN, USA

[3]Purdue University Center for Cancer Research, Purdue University, West Lafayette, IN, USA

Corresponding Authors:

Barbara Stefanska, PhD, MPH
Food, Nutrition and Health Program
Faculty of Land and Food Systems
The University of British Columbia
2205 East Mall
Vancouver, BC V6T 1Z4, Canada
barbara.stefanska@ubc.ca

Sulma Mohammed, PhD, DVM
Department of Comparative Pathobiology
Purdue University Center for Cancer Research
Purdue University
610 Purdue Mall
West Lafayette, IN 47907, USA
mohammes@purdue.edu

*Senior Co-authors

Figure S1. Function and pathway analysis of genes with differentially methylated promoters in atypical ductal hyperplasia (ADH), ductal carcinoma *in situ* (DCIS), and invasive breast cancer compared to normal breast tissue. Kyoto Encyclopedia of Genes and Genomes (KEGG) pathway and Gene Ontology (GO) function analysis was performed using DAVID Knowledgebase. Bars in blue represent pathways enriched with hypomethylated genes. Bars in red represent pathways enriched with hypermethylated genes.

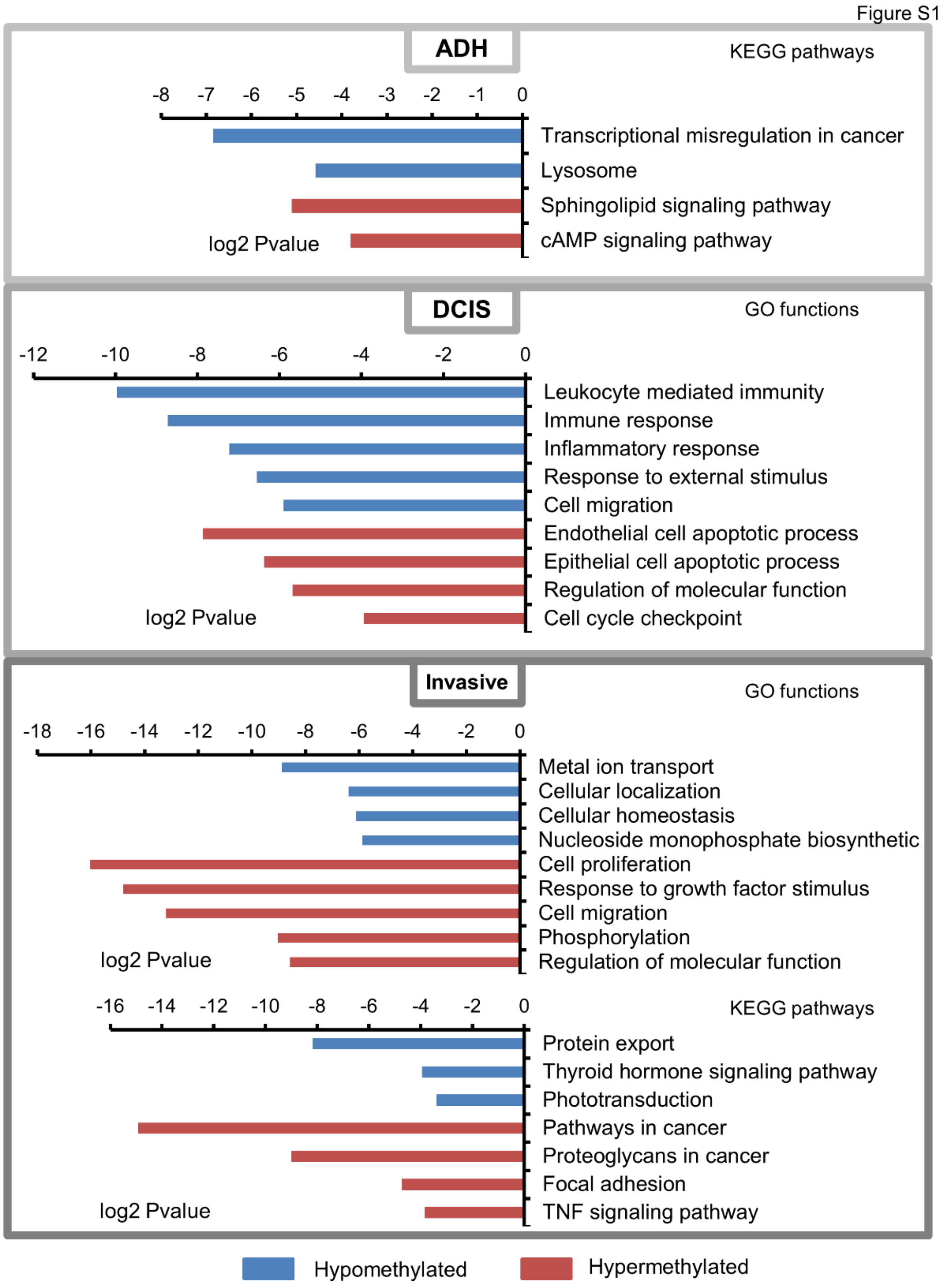

Table S1. Lists of differentially methylated genes from each analysis (invasive/DCIS, invasive only and DCIS only), as measured by Illumina base-calling software.

Hypermethylated gene promoters in DCIS and Invasive							
Gene name	DiffMeth DCIS	p-value DCIS	DiffMeth Invasive	p-value Invasive	Normal	DCIS	Invasive
CTXN1	0.10957	0.013462	0.43398466	3.213E-07	0.510233	0.619805	0.944218
KLK7	0.17997	0.04816	0.42042607	0.0016088	0.500000	0.679971	0.920426
MATR3	0.10772	0.00088763	0.2922183	0.00008548	0.240000	0.347718	0.532218
DNAJC25	0.17362	0.0019877	0.25002507	0.00022908	0.108011	0.281626	0.358036
MCPH1	0.27434	0.0025884	0.20833333	0.00039753	0.625000	0.899342	0.833333
TBX4	0.34952	1.7447E-14	0.18087566	0.0024956	0.378284	0.727806	0.559160
SSR4	0.33652	1.3724E-08	0.16539639	0.000036574	0.122947	0.459462	0.288343
CALCA	0.043753	2.9035E-07	0.16126573	2.5799E-16	0.142827	0.186580	0.304092
CA6	0.078405	0.041433	0.11591036	0.000031336	0.759804	0.838209	0.875714
FASTK	0.061262	4.377E-07	0.09734573	0.00027954	0.216239	0.277502	0.313585
KDR	0.03388	0.0020766	0.09147255	1.3405E-08	0.116720	0.150601	0.208193
HSF4	0.032845	0.034451	0.07073769	5.649E-08	0.281035	0.313880	0.351772
TRIB2	0.005079	0.037702	0.05800376	0.00016157	0.107805	0.112884	0.165809
CTSL2	0.00023382	0.006001	0.05474905	0.00012526	0.242809	0.243043	0.297558
COL7A1	0.19693	6.773E-08	0.04300489	0.021718	0.305933	0.502859	0.348937
CDKN2B	0.044739	0.0042569	0.04135931	0.019348	0.098040	0.142779	0.139399
PODXL	0.01012	0.028657	0.03358135	0.0019467	0.137847	0.147967	0.171429

Hypomethylated gene promoters in DCIS and Invasive							
Gene name	DiffMeth DCIS	p-value DCIS	DiffMeth Invasive	p-value Invasive	Normal	DCIS	Invasive
PFDN1	-0.66667	0.033483	-0.65289256	0.0045568	1.000000	0.333333	0.347107
ATOX1	-0.31323	0.00025312	-0.42095921	0.0081058	0.767196	0.453967	0.346237
CASP9	-0.30038	0.00011296	-0.38870968	0.000001021	0.500000	0.199622	0.111290
SLC7A9	-0.30784	2.0613E-19	-0.37982197	6.4274E-13	0.617475	0.309635	0.237653
OBP	-0.34975	0.019945	-0.28373016	0.040104	0.875000	0.525247	0.591270
TAPBP	-0.3458	3.1314E-08	-0.2640625	0.0003217	0.850000	0.504202	0.585938
FXYD2	-0.1544	0.011847	-0.21384555	3.689E-07	0.881064	0.726659	0.667218
PDE6G	-0.22995	0.036941	-0.19036783	0.047986	0.899306	0.669357	0.708938
CALCB	-0.25878	1.0544E-12	-0.18825507	0.0020299	0.530671	0.271893	0.342416
MADCAM1	-0.31814	9.3449E-10	-0.16912133	0.0030289	0.709375	0.391238	0.540254
OOEP	-0.20106	8.068E-13	-0.14391407	5.4522E-11	0.764549	0.563494	0.620635
SFRP2	-0.12412	0.0032574	-0.14027051	0.00078683	0.432712	0.308587	0.292442
RPL18	-0.19964	3.6527E-10	-0.10513329	2.1817E-07	0.388055	0.188418	0.282922

SEC61G	-0.075099	0.043429	-0.10301565	0.042178	0.211082	0.135984	0.108067
PKD1	-0.23268	6.5256E-08	-0.09860497	0.007912	0.783688	0.551007	0.685083
BDNF	-0.2905	2.2965E-18	-0.05739193	0.017243	0.846138	0.555637	0.788746
PDE6B	-0.15556	4.0418E-10	-0.04375635	0.000067508	0.589329	0.433767	0.545573
RPL31	-0.069089	0.000039452	-0.00561685	0.0022746	0.185185	0.116096	0.179568

Hypermethylated gene promoters in Invasive only				
Gene name	DiffMeth Invasive	p-value Invasive	Normal	Invasive
OTUB1	0.36041656	3.956E-07	0.229730	0.590146
HGF	0.29843296	0.00010256	0.174670	0.473103
VASP	0.28479197	0.0028177	0.117647	0.402439
NKX2-1	0.24605918	0.01	0.182796	0.428855
SSBP1	0.24340799	0.046456	0.093013	0.336421
CLN3	0.24274332	0.017706	0.150775	0.393519
NKX2-5	0.230112	1.0548E-06	0.233563	0.463675
DNASE1	0.22727387	0.0024852	0.762522	0.989796
PPT1	0.22469555	0.037771	0.238095	0.462791
MAGEB10	0.22252747	0.00079211	0.750000	0.972527
SUMO2	0.22049005	0.000078961	0.184397	0.404887
CASP3	0.19178465	0.03331	0.129545	0.321330
HSD17B14	0.18889287	0.002695	0.134146	0.323039
TAF13	0.18815913	0.014999	0.184223	0.372382
HSPA4	0.16623204	1.7921E-07	0.066239	0.232471
PSMD4	0.16195744	0.049756	0.112765	0.274722
LHX3	0.16128399	0.005854	0.228659	0.389943
SGSH	0.15912698	0.00089833	0.325000	0.484127
CNBP	0.15784455	0.000022858	0.182367	0.340212
CDC25A	0.15595991	0.0021698	0.192828	0.348788
DLC1	0.15424718	0.003466	0.079167	0.233414
TEKT1	0.15347061	0.016382	0.184942	0.338413
PDGFRB	0.14629576	0.0053229	0.417087	0.563383
ERBB2	0.14537949	0.040623	0.184211	0.329590
FUNDC2	0.14093949	0.040696	0.339111	0.480051
PDGFB	0.14087712	0.00044825	0.138876	0.279754
RPS6	0.13845429	0.00036808	0.149493	0.287948
C6H16orf93	0.13204966	0.0062201	0.109848	0.241898
CTSD	0.13047831	0.017184	0.167249	0.297727
CEACAM23	0.12483333	0.0055272	0.393305	0.518138
SHOX	0.11675468	0.00072763	0.145521	0.262275
MCHR1	0.11247744	0.002181	0.194161	0.306638

F2RL1	0.10862265	0.011618	0.144432	0.253054
SLC35C1	0.10811519	0.0012024	0.213387	0.321502
TIMM8A	0.10612203	0.031328	0.361772	0.467895
RHPN2	0.10585906	0.0017253	0.166391	0.272250
B3GALT6	0.10482163	6.6211E-08	0.135554	0.240376
PTGES	0.10380291	0.04114	0.684028	0.787831
SERPINH1	0.10144705	0.00015558	0.186062	0.287509
EIF2AK1	0.10069444	0.046425	0.100000	0.200694
NDUFB6	0.09569161	0.042963	0.053150	0.148842
FZD6	0.09405085	0.010397	0.190972	0.285023
FLT3LG	0.09346112	0.0011998	0.278439	0.371900
FXN	0.09215674	0.0096655	0.102273	0.194429
HTR2A	0.0916414	0.0013723	0.112402	0.204043
C3H4orf52	0.08975745	0.019038	0.163846	0.253604
GNA11	0.08837131	0.00003219	0.166358	0.254729
TBX2	0.08139632	0.013779	0.150243	0.231639
VEGFA	0.08112653	0.0005634	0.144822	0.225949
SLC39A7	0.07688904	0.018937	0.144182	0.221071
RECK	0.07463768	0.024221	0.025362	0.100000
MUC1	0.07325877	0.0001096	0.190507	0.263765
CYBA	0.07201556	0.0010351	0.316859	0.388874
ST8SIA2	0.07074791	0.0029548	0.167063	0.237811
CFTR	0.06637243	0.029191	0.159903	0.226276
KCNC1	0.06503805	0.00020648	0.142458	0.207496
GUCY1B3	0.06482928	5.5108E-06	0.134722	0.199552
MCL1	0.06287244	0.027037	0.115789	0.178662
PGR	0.05983644	0.0015508	0.215582	0.275419
PMEL	0.05942142	0.008822	0.180781	0.240202
DZIP1	0.0571715	0.031585	0.175120	0.232291
IDS	0.05609043	0.03241	0.353514	0.409605
DAPK1	0.05548162	0.000013662	0.103167	0.158649
ST13	0.05548128	0.000015413	0.116950	0.172431
NUDT3	0.05177373	0.0035875	0.157756	0.209530
STAR	0.04986375	0.00039644	0.190000	0.239864
TMEM185B	0.04883975	0.01025	0.113249	0.162089
CXCR4	0.0484987	0.0021432	0.133803	0.182302
SLC2A4	0.04571489	0.00071148	0.145674	0.191389
LUC7L2	0.04315298	0.0024977	0.116360	0.159513
TBPL1	0.0429138	0.010756	0.135471	0.178384
CYP1B1	0.03847724	0.000051593	0.104561	0.143038
SOCS3	0.03770657	0.017619	0.162851	0.200558

TJP3	0.03496823	0.00055524	0.296726	0.331694
KHK	0.03345009	0.000069892	0.197281	0.230731
PIM1	0.0321359	0.012157	0.101915	0.134051
GAD2	0.03172593	0.049269	0.216800	0.248526
GPX3	0.03000486	0.02493	0.309524	0.339529
EDNRB	0.02717195	0.00093473	0.194253	0.221425
EPB41L5	0.02492818	0.039495	0.123173	0.148102
KCNJ2	0.02429409	0.01069	0.136169	0.160463
TPM3	0.01879024	0.00024772	0.102179	0.120969
MAPK14	0.0143868	0.040489	0.113734	0.128120
CCS	0.01335358	0.0019013	0.152058	0.165412
CLNS1A	0.00144646	0.0064192	0.197799	0.199245

Hypomethylated gene promoters in Invasive only				
Gene name	DiffMeth Invasive	p-value Invasive	Normal	Invasive
CXCR3	-0.4030303	0.00014861	0.900000	0.496970
TXNRD1	-0.29319869	0.037401	0.461538	0.168340
UCP2	-0.23482906	0.0041639	0.900000	0.665171
TRAF5	-0.22026014	0.0082299	0.822222	0.601962
FUT5	-0.2185915	5.7127E-07	0.786268	0.567677
DIO3	-0.1510582	0.00090755	0.260269	0.109211
SRP19	-0.12109068	0.000057215	0.369605	0.248515
DHRS4	-0.0727794	0.0094245	0.257566	0.184787
FXYD1	-0.05128205	0.048933	0.666667	0.615385
PRF1	-0.04166667	0.0076328	0.875000	0.833333
BEST1	-0.26766612	0.0053329	0.775358	0.507692

Hypermethylated gene promoters in DCIS only				
Gene name	DiffMeth DCIS	p-value DCIS	Normal	DCIS
KCNJ12	0.36477	7.0516E-26	0.186184	0.550958
HOXA10	0.33358	9.3302E-06	0.438095	0.771672
RAB9A	0.2425	0.016516	0.118271	0.360767
MTPN	0.18624	0.00049597	0.155172	0.341414
BIRC5	0.16696	0.011659	0.083333	0.250292
NGB	0.15412	0.023259	0.253292	0.407413
TPC3	0.15398	0.00000183	0.061224	0.215205
COL9A2	0.15289	0.0018253	0.076840	0.229730
BIRC5	0.14312	0.017613	0.083333	0.226455

POR	0.14168	0.0019691	0.758242	0.899923
OAZ1	0.087521	0.0010062	0.114667	0.202188
SPCS2	0.086802	0.0025919	0.082418	0.169220
HMOX1	0.084614	0.020503	0.161399	0.246013
OXTR	0.062907	0.0015108	0.183877	0.246784
RPL27A	0.060934	0.025615	0.085775	0.146709
XYLT1	0.051022	0.027843	0.218568	0.269589
IL13	0.04864	0.000058652	0.269841	0.318482
ARHGEF2	0.02648	0.00087061	0.616667	0.643147
KCNA2	0.024567	0.022257	0.200000	0.224567
PSMC1	0.023366	0.018318	0.243243	0.266609
C15H16orf87	0.023227	0.0064624	0.072985	0.096212
ADRB2	0.0046515	0.020005	0.189529	0.194181
C16H7orf55	0.0010118	0.04186	0.267401	0.268412

**Hypomethylated gene promoters in DCIS only**				
Gene name	DiffMeth DCIS	p-value DCIS	Normal	DCIS
COL4A5	-0.45434	0.000076202	0.77273	0.318386
GUCY1A3	-0.37588	0.000051671	0.555556	0.179675
LOC448801	-0.33604	2.2866E-27	0.813514	0.477476
TAS2R3	-0.31753	0.029667	0.928571	0.611039
TPO	-0.27733	2.242E-42	0.783068	0.505736
MS4A2	-0.25052	0.000018604	0.885380	0.634855
TH	-0.24339	0.0029159	0.843254	0.599862
GTF2A1L	-0.23219	2.4853E-07	0.947917	0.715722
CCL1	-0.2254	0.0088979	0.825397	0.600000
MAOB	-0.22213	0.0018207	0.494118	0.271991
PRR5	-0.2144	4.5567E-09	0.463492	0.249095
TLR9	-0.18819	0.026256	0.604167	0.415976
STOM	-0.18423	0.039658	1.000000	0.815774
TNNT2	-0.17378	0.023855	0.871429	0.697651
VMA21	-0.15676	0.040684	0.470588	0.313826
CAPS	-0.1183	0.014268	0.572704	0.454406
MYH3	-0.1143	0.029671	0.746569	0.632273
PMAIP1	-0.11134	0.0022644	0.541033	0.429689
PPBP	-0.10246	0.00032863	0.699123	0.596666
MT1E	-0.098785	0.0041606	0.193111	0.094325
NDUFS8	-0.085565	0.035923	0.300647	0.215082
SEC61A1	-0.082632	0.018131	0.277778	0.195146
ANXA4	-0.078468	0.018784	0.964809	0.886341

AP3S2	-0.069672	0.046983	0.156428	0.086756
GSTA4	-0.069473	0.034738	0.200000	0.130527
TGM1	-0.061503	0.041364	0.477315	0.415812
PLA2G7	-0.051595	0.044361	0.203660	0.152065
EIF4A1	-0.040918	0.043752	0.143237	0.102319
AKR1A1	-0.039775	0.033625	0.206944	0.167169
PMEL	-0.038829	0.0095114	0.180781	0.141952
IL15	-0.037622	0.00013217	0.945520	0.907898
BDKRB1	-0.035857	0.0019114	0.755952	0.720096
STXBP2	-0.034595	0.0011115	0.387500	0.352905
RPL23	-0.034531	0.010036	0.226085	0.191553
TMED11	-0.031804	0.019318	0.903095	0.871291
LGALS1	-0.02472	0.02984	0.230819	0.206100
FBXO9	-0.021642	0.044492	0.087585	0.065943
DLA-12	-0.019502	0.021764	0.231041	0.211538
SNAP91	-0.018634	0.012015	0.177593	0.158959
CLN5	-0.017636	0.025979	0.127273	0.109636
CD8A	-0.0049317	0.043925	0.544643	0.539711
FOSB	-0.0026634	0.04606	0.140512	0.137849

Table S2. Table of primer sequences used for qPCR analysis.

**Supplementary Table S2**. Primer sequences used in qPCR analysis.			
**Gene**	**Primer sequences**	**Annealing temperature [°C]**	**Amplicon length [bp]**
**qPCR**			
GAPDH	FW 5'-TGCACCACCAACTGCTTA-3'<br>RV 5'-AGAGGCAGGGATGATGTTC-3'	59	177
CASP3	FW 5'-CTCTGGAATATCCCTGGACAAC-3'<br>RV 5'-ACATCTGTACCAGACCGAGA-3'	59	123
CTXN1	FW 5'-CCACCTTGACTGAGTGTTGA-3'<br>RV 5'-GTGCAGGGCAAATTGGTATG-3'	59	102
COL7A1	FW 5'-CTGATCACAGACGGGAAGTC-3'<br>RV 5'-CTCAGGGTCAGCATTCTTGAT-3'	59	108
RECK	FW 5'-ACTGCCGAGAATACTGTCAAG-3'<br>RV 5'-GTGGACTAATAGAGGCGCAATA-3'	59	99
SSBP1	FW 5'-CCCTGAATCGTGTGCACTTA-3'<br>RV 5'-CCTGATCGCCACATCTCATT-3'	59	118
MCPH1	FW 5'-ACACAGCTTGTGGATATGGG-3'<br>RV 5'-GTGCTCTGGTAGCCATCTTT-3'	59	89

IDS	FW 5'-CTGTGGATGTGCTGGATGTT-3'<br>RV 5'-GGGATGTGTGGCTTATGATACC-3'	59	139
DNASE1	FW 5'-CGTGTCCCTGAAGATCGC-3'<br>RV 5'-TGCACAATGTAGCTGACGAG-3'	59	87
FUNDC2	FW 5'-ACTGGACCTTGCGGAATTT-3'<br>RV 5'-GGGTTGCCACGCTATACTT-3'	59	98
FZD6	FW 5'-TGGCCTACAACATGACGTTT-3'<br>RV 5'-CCAGATTTGCGAGAGGAAGAA-3'	59	99
